# Iron and peroxide regulation of the PrrF sRNAs and a conserved ferritin family protein in *Pseudomonas aeruginosa* and *Pseudomonas fluorescens*

**DOI:** 10.1128/jb.00534-25

**Published:** 2026-03-30

**Authors:** Khady O. Ouattara, Rhishita Chourashi, Amanda G. Oglesby

**Affiliations:** 1 Department of Pharmaceutical Sciences, School of Pharmacy, University of Maryland 15513, Baltimore, Maryland, USA; 2 Department of Microbiology and Immunology, School of Medicine, University of Maryland 15513, Baltimore, Maryland, USA; Dartmouth College Geisel School of Medicine, Hanover, New Hampshire, USA

**Keywords:** PrrF, BrnD, *Pseudomonas aeruginosa*, *Pseudomonas fluorescens*, iron, oxidative stress, Dps

## Abstract

*Pseudomonas aeruginosa* is a gram-negative opportunistic pathogen that requires iron to cause infection. Iron can also be toxic due to its participation in Fenton chemistry, resulting in the production of reactive oxygen species (ROS). Thus, *P. aeruginosa* regulates the uptake, use, and storage of iron to mitigate the effects of ROS. *P. aeruginosa* uses several mechanisms to manage oxidative stress, including superoxide dismutases, catalases, and members of the ferritin superfamily. The iron-responsive PrrF1 and PrrF2 small regulatory RNAs (sRNAs) are predicted to pair with and destabilize mRNA transcripts for several oxidative stress response proteins, including *sodB*, *katA*, and *brnD*, encoding a novel bacterioferritin-like Dps protein. In this study, we developed *brnD* reporter constructs that are responsive to PrrF-mediated iron regulation. We demonstrated that PrrF-mediated regulation of *brnD* occurs in the 5′ untranslated region of its mRNA, likely via a conserved region of complementarity with the PrrF sRNAs. We further showed that *brnD* mRNA levels are increased upon hydrogen peroxide treatment. Surprisingly, peroxide treatment also resulted in elevated levels of the PrrF sRNAs. Last, we investigated these regulatory effects in *Pseudomonas fluorescens*, revealing similar iron regulation of the PrrF sRNAs and *brnD* ortholog, as well as peroxide-induced expression of the PrrF sRNAs. Combined, these data highlight a distinct class of iron-responsive ferritin family proteins with potential functional conservation across the pseudomonads, and they reveal a novel aspect of the oxidative stress response involving the PrrF sRNAs.

**IMPORTANCE:**

Iron is a crucial micronutrient for *Pseudomonas aeruginosa* survival and virulence, yet it can also be toxic due to the production of reactive oxygen species. The PrrF small regulatory RNAs (sRNAs) are transcribed in iron-limiting conditions and block the expression of several mRNAs involved in *P. aeruginosa*’s oxidative stress response. Here, we make the surprising discovery that oxidative stress induces expression of the PrrF sRNAs. We also provide evidence that PrrF regulation of two mRNA targets is hindered upon oxidative stress. Oxidative stress similarly induces the PrrF sRNAs in *Pseudomonas fluorescens*, indicating conservation of this novel regulatory event. This study, therefore, highlights a novel and conserved regulatory link between iron homeostasis and oxidative stress protection in the pseudomonads.

## INTRODUCTION

Iron is required for the survival of almost all organisms but can also be toxic due to its reactivity with oxygen. Through Fenton chemistry, iron catalyzes the formation of the hydroxyl radical, which can react with proteins, lipids, and DNA to cause cellular damage. Aerobic organisms, therefore, employ numerous proteins to detoxify reactive oxygen species (ROS), including superoxide dismutases, catalases, and alkyl hydroperoxidase reductases (1, 2). Organisms also use members of the ferritin superfamily to protect against the effects of iron-mediated oxidative stress. An important facet of these ferritin-like proteins is their ferroxidase centers that oxidize iron from the ferrous [Fe(II)] to the ferric [Fe(III)] ion, which is stored as an insoluble mineral in the protein core (3, 4). Members of the ferritin superfamily include ferritins (Ftn), bacterioferritins (Bfr), and DNA-binding protein from stationary-phase cells (Dps) proteins. Ftns are conserved across all domains of life, while Bfr and Dps proteins are specific to prokaryotes. Ftns and Bfrs both form 24-mers that function primarily as iron storage proteins, and Bfrs are differentiated from Ftns by a methionine residue that coordinates heme (5). In contrast to Bfrs and Ftns, Dps proteins form 12-mers that, in some organisms, bind to and protect DNA when the cell is starved for nutrients (6–8). Because of the conservation of ferritin proteins, understanding the mechanisms by which different ferritin superfamily proteins contribute to iron homeostasis may be broadly translated to multiple organisms.

**Fig 1:**
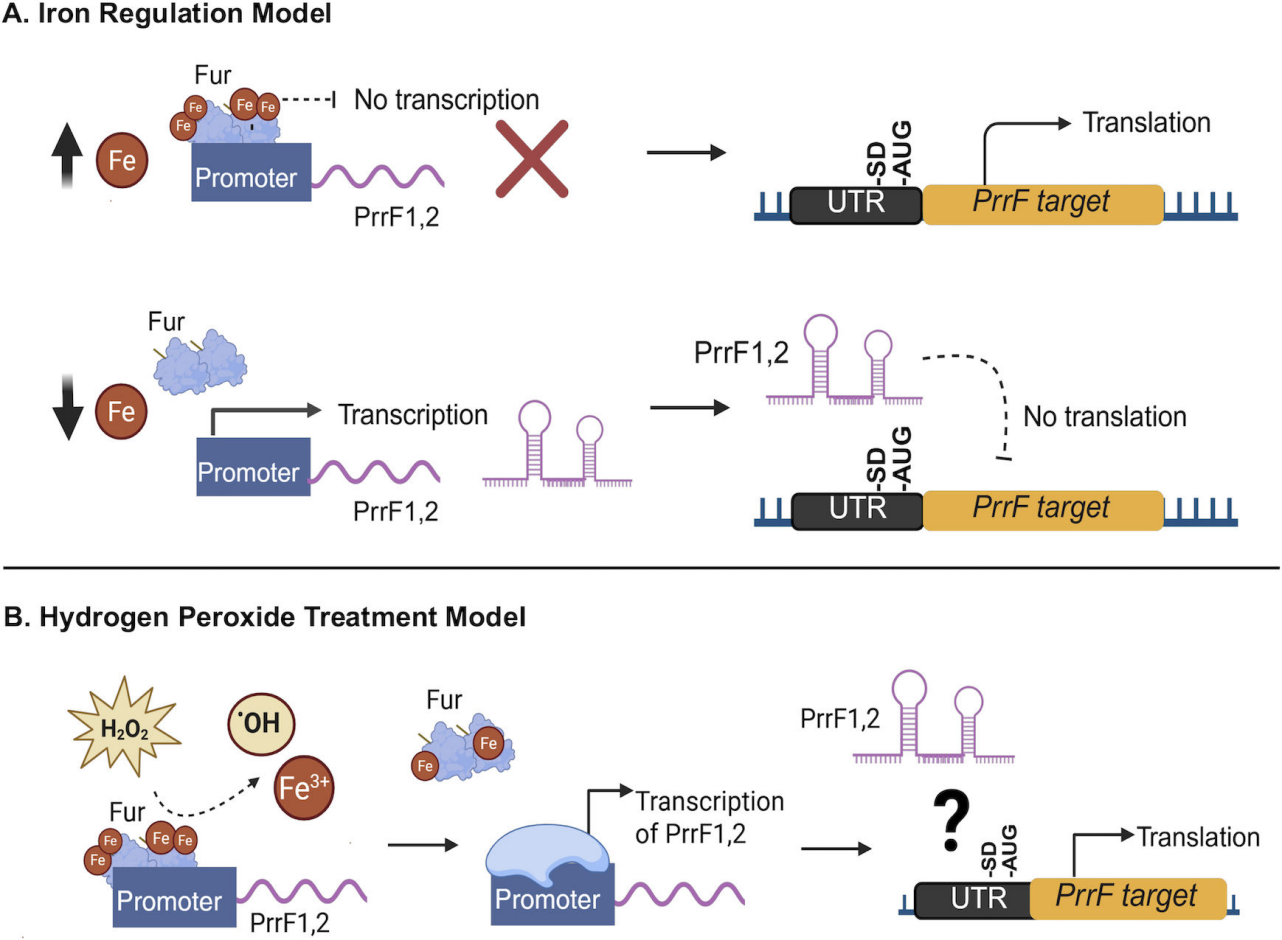
Model of iron and oxidative stress regulation of PrrF and *brnD*. (**A**) PrrF post-transcriptionally represses PrrF targets during iron starvation. (**B**) Hydrogen peroxide results in the induction of the PrrF sRNAs and perturbs PrrF regulation of *brnD* and *sodB*.

*Pseudomonas aeruginosa* is a ubiquitous environmental bacterium that causes multi-drug-resistant infections in compromised individuals, including those with the hereditary disease cystic fibrosis (CF). *P. aeruginosa* requires iron for growth and infection, yet the mammalian host limits this nutrient during infection through a process referred to as nutritional immunity (9). During infection, *P. aeruginosa* overcomes host-mediated iron limitation through the expression of multiple high-affinity iron uptake systems. *P. aeruginosa* also mitigates the potential for iron-mediated oxidative stress, which can be exacerbated via the production of ROS by the host immune system. To prevent excess iron accumulation, expression of iron uptake genes is regulated by the ferric uptake repressor (Fur) protein, which blocks transcription of these genes when it is bound to cytosolic iron [Fe(II)]. Fur also represses expression of the PrrF1 and PrrF2 small noncoding regulatory RNAs (sRNAs) (10, 11). The PrrF sRNAs mediate an iron-sparing response by pairing with and decreasing the stability and translation of mRNAs encoding non-essential iron-containing proteins (10, 12, 13). The resulting regulation allows for expression of PrrF-regulated genes when cytosolic iron levels are sufficient, while repressing expression of iron-dependent pathways when this nutrient is limiting (Fig. 1A). The PrrF sRNAs are required for acute murine lung infection, and their expression is conserved in clinical isolates from CF sputa (11, 13). However, our understanding of how the PrrF sRNAs contribute to *P. aeruginosa* virulence across different disease models remains incomplete, particularly how PrrF regulation of specific targets contributes to fitness in varying environments.

The PrrF regulon consists of several mRNAs encoding ROS detoxifying and iron storage proteins. Among these are SodB, an iron-cofactored superoxide dismutase that converts the superoxide radical into oxygen and hydrogen peroxide (H_2_O_2_), and the heme-cofactored catalase, KatA, which converts H_2_O_2_ into water and oxygen (14, 15). PrrF also negatively affects the expression of, and shares complementarity with, an mRNA that is annotated as a probable Bfr (10). The annotation arose from conserved Bfr ferroxidase center residues as well as a potential heme-coordinating methionine residue (5, 16). However, recent structural studies of PA4880 revealed that it complexes as a 12-mer, suggestive of a Dps protein, while also possessing a ferroxidase center that is structurally similar to those formed by Bfrs (17). This study further found no evidence of PA4880 interacting with heme (17), and the heme-coordinating methionine residue is not conserved in orthologs from other *Pseudomonas* species (Fig. S1). Prior studies characterized two different ferritin family proteins that share these structural features and named the proteins DpsL for “Dps-like” (18–20). However, a broader literature search revealed that the term “Dps-like” has been generically used to describe numerous Dps-related proteins that, based on gene sequence, do not share PA4880 and the related proteins’ structural features (21–25). Moreover, phylogenetic analysis of ferritin family proteins classified these two DpsL proteins as an outgroup of Dps proteins, indicating that they preceded the evolution of either Bfr or Dps and should be considered part of a distinct sub-family (26). We therefore propose renaming PA4880 and these two related proteins as bacterioferritin-like Dps proteins, or BrnD, to convey more specificity and avoid confusion in the literature. While a phenotype for the *P. aeruginosa* ∆*brnD* mutant has not yet been identified, the conservation of this gene across *Pseudomonas* species suggests that it plays an important role in these organisms. Notably, *P. aeruginosa* also encodes a bacterioferritin (BfrB), ferritin (FtnA), and Dps protein (PA0962) (27), yet only BrnD is regulated by PrrF (10, 12), indicating a link with iron homeostasis.

In the current study, we examined how PrrF sRNA-mediated regulation links iron homeostasis with oxidative stress responses, focusing primarily on *brnD*. Using *brnD* reporter strains, we show that PrrF regulates *brnD* through its 5′ untranslated region (UTR), likely via a conserved region of complementarity between PrrF and *brnD*. We also demonstrate that hydrogen peroxide treatment of cultures results in increased *brnD* mRNA levels and, unexpectedly, increased PrrF expression. Moreover, we show similar regulation of *brnD* and PrrF expression in the environmental pseudomonad *Pseudomonas fluorescens*. Together, these findings reveal conserved iron regulation of a distinct class of ferritin family proteins in the pseudomonads and uncover a novel oxidative stress response involving the PrrF sRNAs.

## RESULTS

### Iron activates *brnD* expression in a PrrF-dependent manner

**Fig 2:**
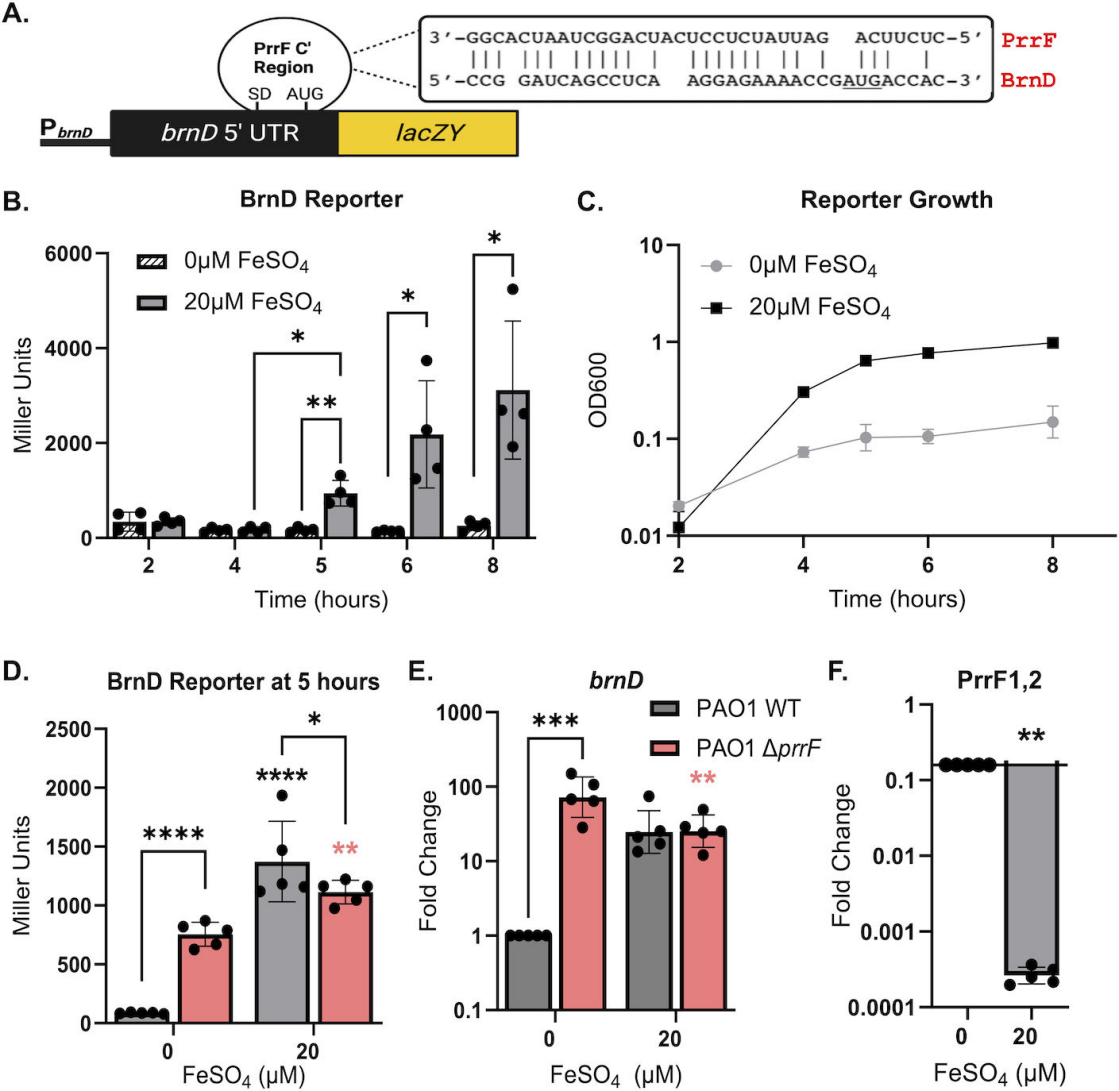
Reporter shows PrrF-mediated iron induction of *brnD*. (**A**) Diagram of the *brnD* reporter construct showing the approximate location of complementarity with the PrrF sRNAs overlapping the Shine-Dalgarno (SD) and translational start site (AUG). (**B and C**) PAO1 carrying the *brnD* reporter construct was grown with shaking (250 rpm) in chemically defined medium (CDM) with or without supplementation of the indicated concentrations of FeSO_4_ over the span of 8 h and assayed for β-galactosidase activity (**B**) and culture density (**C**). (**D–F**) PAO1 (gray bars) and the isogenic Δ*prrF* mutant (pink bars) carrying the *brnD* reporter were grown for 5 h and analyzed for β-galactosidase activity (**D**), and RNA was isolated for qPCR analysis of the *brnD* mRNA (**E**) and the PrrF sRNAs (**F**). Statistics were performed using two-way analysis of variance (ANOVA), with Tukey’s multiple comparisons test for significance. Floating asterisks represent strain-specific (black for wild-type and pink for Δ*prrF*) significance in reference to 0 μM FeSO_4_, to which all conditions were normalized: ****P < 0.0001, ***P < 0.0005, **P < 0.005, and *P < 0.05.

Previous transcriptomic studies showed that *brnD* expression is negatively affected by the PrrF sRNAs in iron-depleted conditions (10, 12). To further investigate how PrrF and iron regulate *brnD* expression, we constructed an expression reporter with the *brnD* promoter and 5′ UTR fused to *lacZ* (Fig. 2A). The resulting reporter construct was introduced into the CTX phage attachment site on the chromosomes of *P. aeruginosa* PAO1 and an isogenic Δ*prrF* mutant lacking the *prrF1* and *prrF2* genes. Since BrnD shares structural features with Dps proteins and is predicted to play a role in oxidative stress that can be exacerbated by high levels of iron, we first aimed to determine the minimum concentration of iron needed to fully de-repress *brnD* reporter activity. For this, the reporter strains were grown in chelexed and dialyzed tryptic soy broth supplemented with ferric chloride (FeCl_3_) at various concentrations (0–100 µM) for 18 h, conditions that have been used for most prior PrrF and iron regulatory studies (11, 28). Analysis of β-galactosidase activity showed that the *brnD* reporter in the wild-type (WT) strain was significantly induced by as little as 20 μM FeCl_3_ (Fig. S2A). Moreover, the ∆*prrF* reporter strain showed significantly increased *brnD* reporter activity in the absence of iron supplementation (Fig. S2B), demonstrating that the *brnD* reporter is subject to PrrF-mediated iron regulation.

We next conducted these experiments using a chemically defined medium (CDM) that we have more recently implemented for metal regulation studies (Fig. S2C and D) (29, 30). The base CDM is supplemented with 1 mM Ca, 0.3 μM Mn, 6 μM Zn, 0.1 μM Ni, and 0.1 μM Cu, concentrations that have previously been reported in CF sputa (31–33), to avoid mis-metallation of proteins upon iron supplementation. We also replaced FeCl_3_ with ferrous sulfate (FeSO_4_), a more soluble iron source, to reduce precipitation of iron salts during culture. Time course analysis of the wild-type *brnD* reporter strain grown in iron-supplemented CDM showed that the *brnD* reporter becomes active after 5 h of growth (Fig. 2B), corresponding with early stationary phase (Fig. 2C). In contrast, the activity of the *brnD* reporter is repressed in the wild-type strain grown in iron-depleted conditions, and this repression is relieved in the Δ*prrF* mutant reporter strain (Fig. 2D). Consistent with analysis of the reporter strains, qPCR showed that *brnD* mRNA levels are similarly de-repressed in the ∆*prrF* mutant compared to wild-type grown in iron-depleted CDM at 5 h of growth (Fig. 2E). While *brnD* mRNA levels were 20-fold higher upon iron supplementation of wild-type cultures in CDM, this induction was not statistically significant (gray bars in Fig. 2E). We also analyzed PrrF sRNA expression by qPCR using oligonucleotides that detect both the PrrF1 and PrrF2 sRNAs (13) and found that supplementation of the medium with 20 µM iron significantly downregulates the PrrF RNAs (Fig. 2F), correlating with increased *brnD* expression. Since CDM is a defined medium, allowing for better control of metals, we used this medium for the remainder of the studies described herein.

### PrrF regulation of *brnD* occurs in static culture

**Fig 3:**
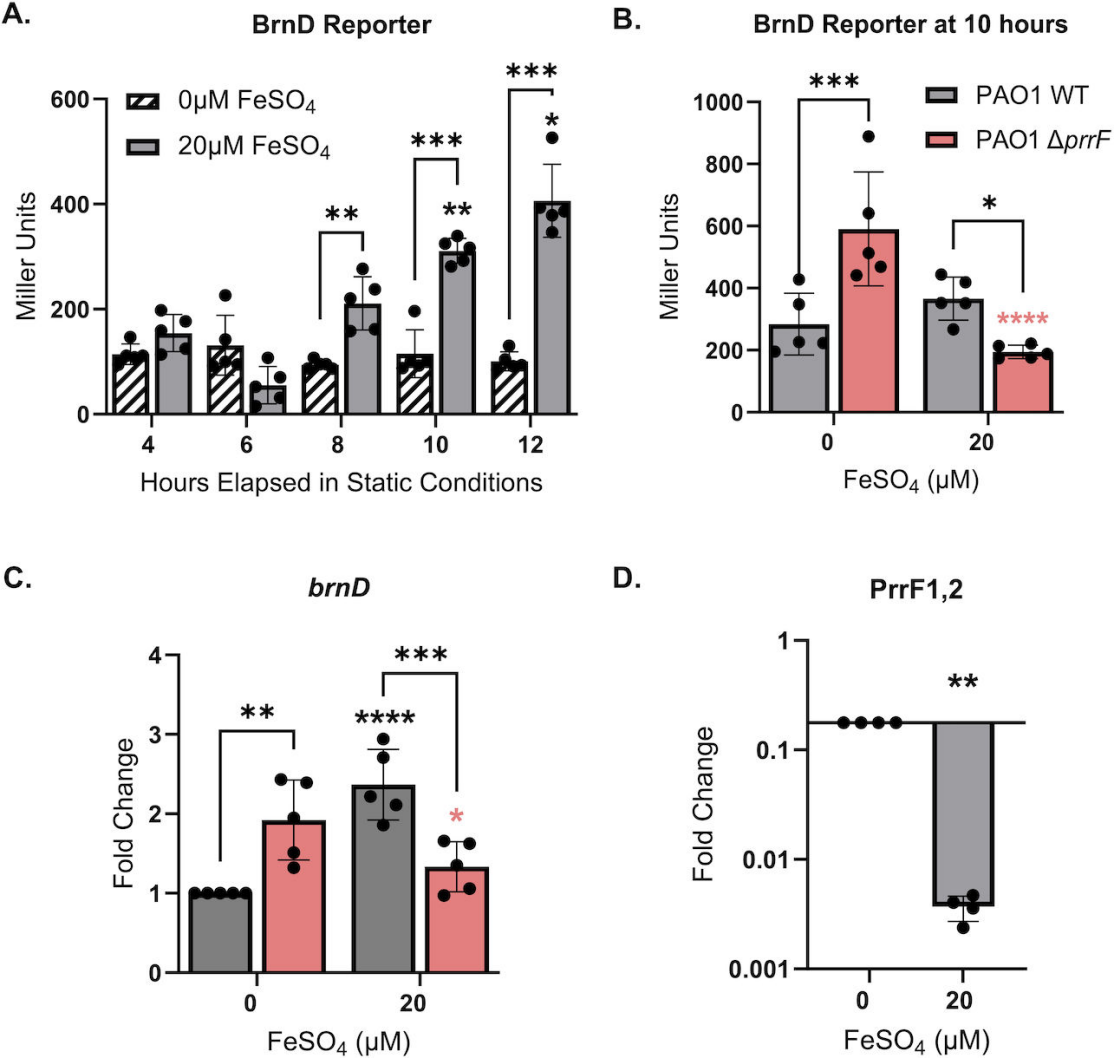
PrrF regulation of *brnD* is conserved in static culture. (**A**) PAO1 (gray bars) carrying the *brnD* reporter construct was grown statically in CDM with or without supplementation of 20 µm FeSO_4_ over the span of 12 h and assayed for β-galactosidase activity. (**B–D**) PAO1 and the isogenic Δ*prrF* mutant (pink bars) strains carrying the *brnD* reporter were grown statically for 10 h and analyzed for β-galactosidase activity (**B**), and RNA was isolated for qPCR analysis of the *brnD* mRNA (**C**) and the PrrF sRNAs (**D**). Statistics were performed using two-way analysis of variance (ANOVA), with Tukey’s multiple comparisons test for significance (**A, B, and D**) and one sample *t*-test for panel **C**. Floating asterisks represent condition-specific significance in reference to the 4 h time point (**A**) or strain-specific significance (black asterisks for wild-type and pink asterisks for Δ*prrF*) in reference 0 μM FeSO_4_ (**B–D**): ****P < 0.0001, ***P < 0.0005, **P < 0.005, and *P < 0.05.

Prior expression studies demonstrated that *brnD* is induced upon hypoxia in an Anr-dependent manner (34) and co-regulated with genes for alginate production that support biofilm formation in the hypoxic CF lung environment (35–37). Since our own work has demonstrated that iron and PrrF regulation are altered in hypoxic and anaerobic environments (30, 38), we were prompted to determine whether PrrF-mediated iron regulation of *brnD* would similarly occur in low oxygen environments. To test this idea, we grew the wild-type and ∆*prrF* mutant reporter strains in CDM for 12 h in static cultures, which exhibit gene expression profiles indicative of hypoxia (38). We observed significant induction of *brnD* reporter activity with 20 µM iron supplementation after 10 h of static growth (Fig. 3A), although we noted that this induction was not consistent from experiment to experiment (Fig. 3B), possibly due to overall weaker reporter activity in static versus shaking cultures. However, qPCR analysis showed consistent two- to fourfold upregulation of the *brnD* mRNA upon supplementation of cultures with 20 µM iron (Fig. 3C). Additionally, *brnD* reporter activity and mRNA levels were significantly de-repressed in the ∆*prrF* mutant compared to the wild-type grown in iron-depleted conditions, indicating that iron induction of *brnD* in the wild-type strain is due to loss of PrrF repression (Fig. 3B and C). Also, as expected, the PrrF sRNAs were significantly repressed by supplementation of static cultures with 20 µM iron after 10 h of growth (Fig. 3D), although not to the same extent as in shaking cultures (Fig. 2E). Taken together, these data indicate that PrrF-mediated iron regulation of *brnD* occurs under hypoxic conditions.

### PrrF regulation of *brnD* mRNA occurs via its 5′ UTR during static growth

**Fig 4:**
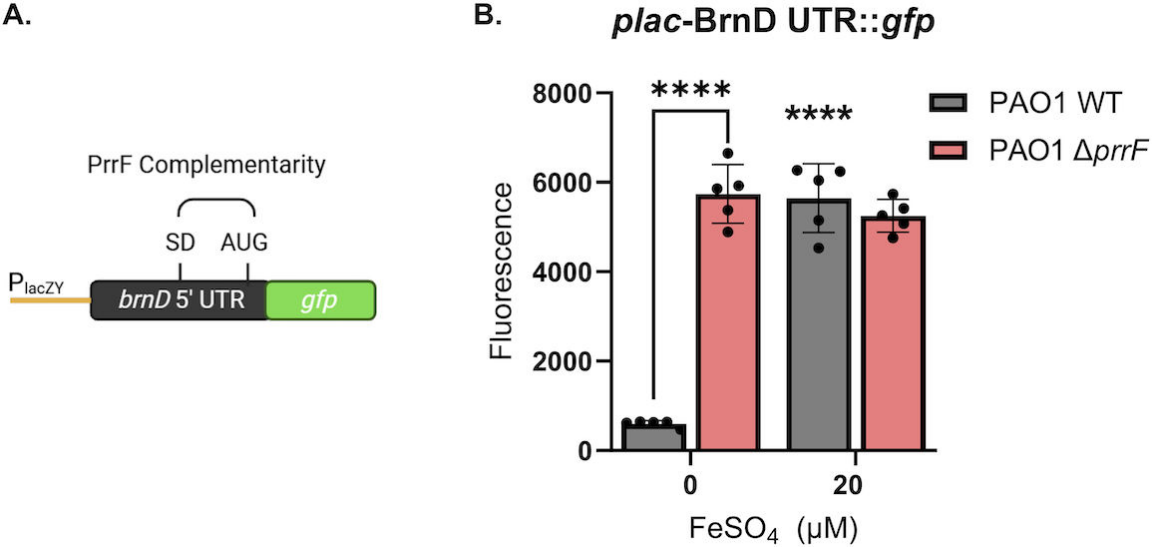
GFP reporter indicates PrrF regulation occurs at the *brnD* 5′ UTR. (**A**) Diagram of the *brnD* translational reporter construct. (**B**) Wild-type (gray bars) and ∆*prrF* (pink) strains carrying the *brnD* translational reporter were grown statically for 10 h in CDM with or without supplementation of 20 μm FeSO_4_. GFP was excited at 485 nm and fluorescence emitted at 528 nm, with a gain of 100. Statistics were done using two-way analysis of variance (ANOVA), with uncorrected Fisher’s least significance difference (LSD) for significance. Floating asterisks represent significance compared to the WT strain in low iron: ****P < 0.0001.

To determine if the 5′ UTR of the *brnD* mRNA is responsible for the observed iron and PrrF regulation of *brnD*, we constructed a translational reporter with the *brnD* 5′ UTR fused to the 3′ end of the P*_lac_* promoter and the 5′ end of *gfp* (Fig. 4A). The resulting construct was introduced into the CTX phage attachment site of PAO1 wild-type and ∆*prrF* strains. While we did not observe robust iron- or PrrF-mediated regulation of fluorescence in shaking cultures (Fig. S3), iron-induced fluorescence of the wild-type strain was observed after 10 h of static growth (Fig. 3B). Moreover, reporter activity was de-repressed in the ∆*prrF* mutant when grown statically in low iron conditions (Fig. 4B). It is unclear why the translational reporter was not responsive to iron or PrrF regulation during shaking growth. Nevertheless, the static culture data are consistent with a model where the PrrF sRNAs pair with the *brnD* mRNA in the 5′ UTR to affect iron regulation of *brnD* expression.

### Peroxide stress increases levels of *brnD* and PrrF RNAs during static growth

**Fig 5:**
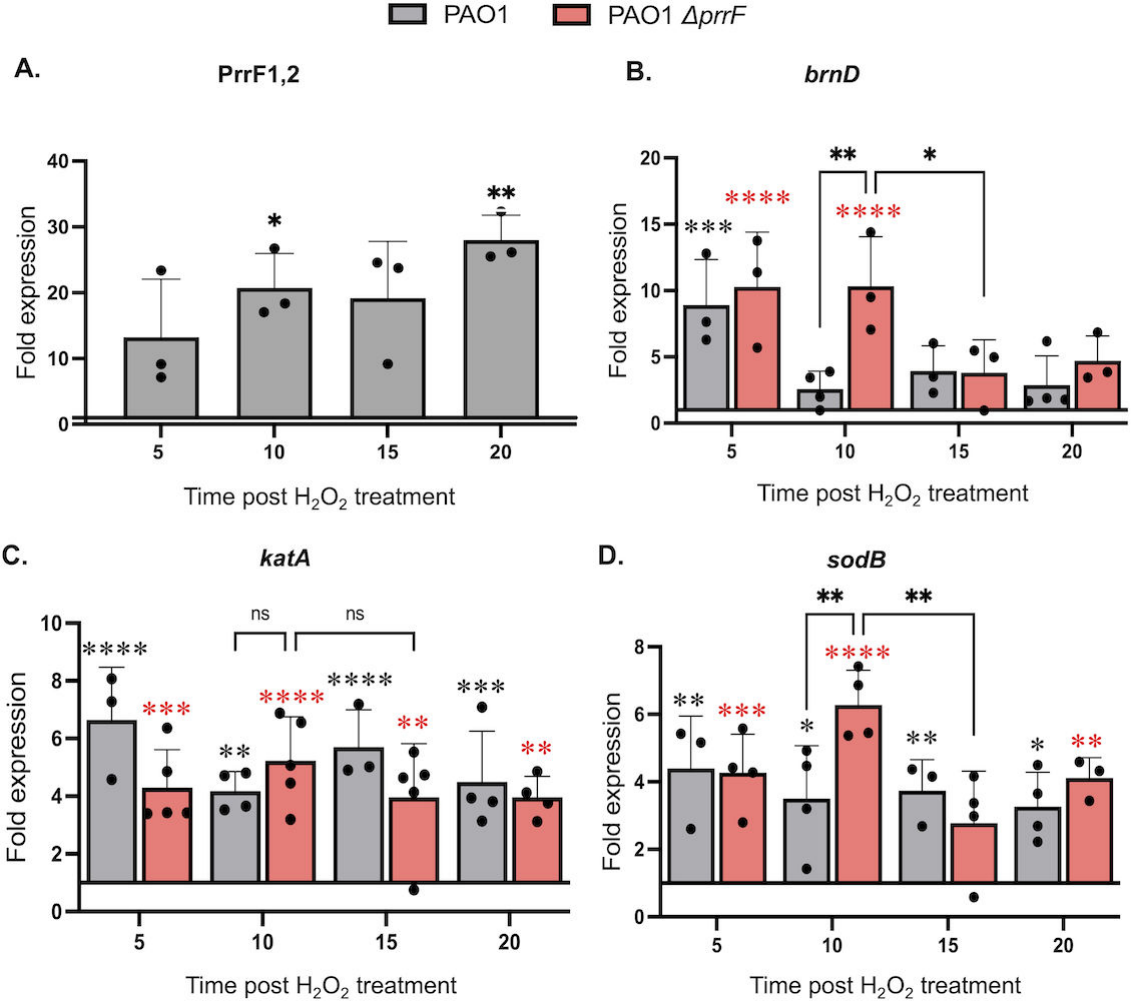
Peroxide stress induces PrrF and its targets in static culture. The indicated strains were grown statically in CDM supplemented with 20 µm FeSO_4_ for 10 h, then treated with 5 mM H_2_O_2_ in static conditions, at which point RNA was isolated and used for qPCR analysis of PrrF (**A**), *brnD* (**B**), *katA* (**C**), and *sodB* (**D**). Statistics were done using mixed effects analysis with Dunnett’s multiple comparisons test for panel **A** and two-way analysis of variance (ANOVA) with Tukey’s multiple comparisons test (**B–D**). Floating asterisks represent strain-specific (black for wild-type and pink for Δ*prrF*) significance in reference to the pre-treatment sample: ****P < 0.0001, ***P < 0.0005, **P < 0.005, and *P < 0.05.

Dps proteins, which share some structural similarity to BrnD, contribute to oxidative stress protection of many organisms (8, 27). Therefore, we next questioned whether *brnD* expression is responsive to oxidative stress. To test this idea, the wild-type and ∆*prrF* strains were grown for 10 h in CDM with 20 µM iron supplementation to promote *brnD* expression, then treated with 5 mM H_2_O_2_. The concentration of H_2_O_2_ was based on prior studies (39–42). The resulting cultures were analyzed for gene expression by qPCR for 20 min post-treatment. Surprisingly, peroxide treatment resulted in significantly increased levels of the PrrF sRNAs, despite cultures being supplemented with iron (Fig. 5A). Peroxide treatment also resulted in increased mRNA levels of *brnD* (Fig. 5B), suggesting that PrrF, although expressed, was inactive as a repressor of *brnD* immediately after oxidative stress. As positive controls for peroxide-mediated oxidative stress, we examined expression of *katA* and *sodB*, which were also induced upon peroxide treatment (Fig. 5C and D). We also noted that, at 10 min post-treatment, *brnD* and *sodB* levels were significantly higher in the ∆*prrF* mutant compared to wild type (Fig. 5B and D). One explanation for these data is that PrrF repression of these two mRNAs resumed in the wild-type strain by 10 min after treatment, while a PrrF-independent mechanism restored levels of these mRNAs to pre-treatment levels at later time points. In contrast, *katA* mRNA levels remained high in both the wild-type and ∆*prrF* mutant throughout the time course (Fig. 5C). Consistent with these data, PrrF negatively affected *sodB* but not *katA* expression during static growth in CDM (Fig. S4A and B). These data reveal that *brnD* expression is responsive to oxidative stress, and they suggest that peroxide treatment disrupts PrrF regulation of *sodB*.

### Peroxide treatment results in upregulation of the *prrF2* promoter

**Fig 6:**
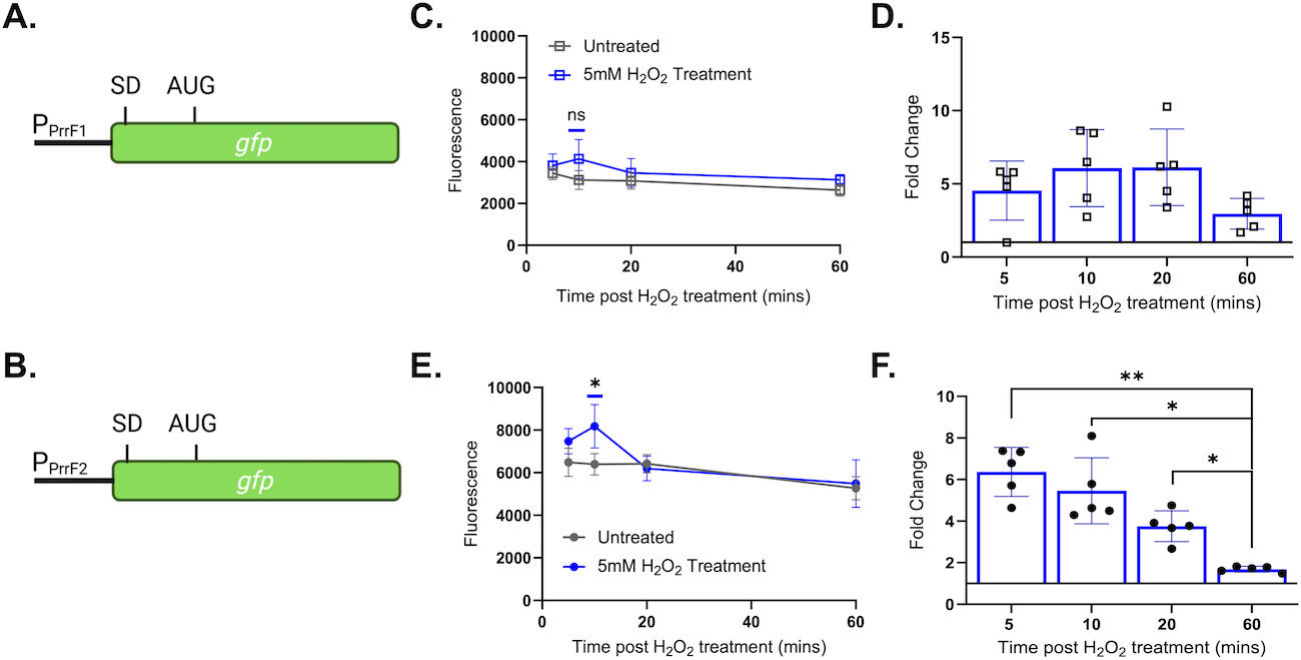
Peroxide differentially induces the *prrF2* promoter. (**A and B**) Diagrams of the P*_prrF1_* (**A**) and P*_prrF2_* (**B**) transcriptional reporter fusions. SD: Shine-Dalgarno. (C–F) PAO1 carrying the P*_prrF1_* (**C and D**) or P*_prrF2_* (**E and F**) transcriptional reporter constructs was grown statically for 10 h in CDM supplemented with 20 μM FeSO_4_ and then treated with 5 mM H_2_O_2_. Aliquots were collected and assayed for fluorescence (**C and E**) or harvested for RNA isolation and qPCR analysis of the *gfp* mRNA (**D and F**) at the indicated timepoints post-treatment. GFP was excited at 485 nm and fluorescence emitted at 528 nm, with a gain of 100. Statistics were performed using two-way analysis of variance (ANOVA), with Tukey’s multiple comparisons test for significance: **P < 0.005 and *P < 0.05.

The Fur protein binds to cytosolic Fe(II)—the predominant ion in the reducing cytosolic environment—to become an active transcriptional repressor (43, 44). A prior study showed that peroxidase and catalase-null *Escherichia coli* mutants exhibited oxidized cytosols, which resulted in deactivation of Fur and de-repression of Fur targets (41). We therefore questioned if increased PrrF sRNA levels upon peroxide treatment were similarly due to Fur inactivation. For this, we determined whether H_2_O_2_ treatment leads to increased *prrF* transcription initiation using reporter constructs with either the *prrF1* or the *prrF2* promoters fused to *gfp* (Fig. 6A and D). The *prrF* reporter strains were grown in CDM supplemented with 20 µM iron for 10 h, then treated with 5 mM H_2_O_2_. While we observed an increase in both fluorescence and *gfp* mRNA levels upon treatment of the *prrF1* reporter strain, neither of these increases was statistically significant (Fig. 6B and C). In contrast, the *prrF2* reporter strain showed significant increases in both fluorescence and *gfp* mRNA levels upon H_2_O_2_ treatment (Fig. 6E and F). We interpret the increase in PrrF sRNA levels to be partially due to reduced Fur activity. Due to the insignificant increase in *prrF1* promoter activity, it remains unclear if PrrF induction is specific to *prrF2* or if the power of the experiment (*n* = 5) is too low.  

### Iron regulation of *brnD* is conserved in *P. fluorescens*

**Fig 7:**
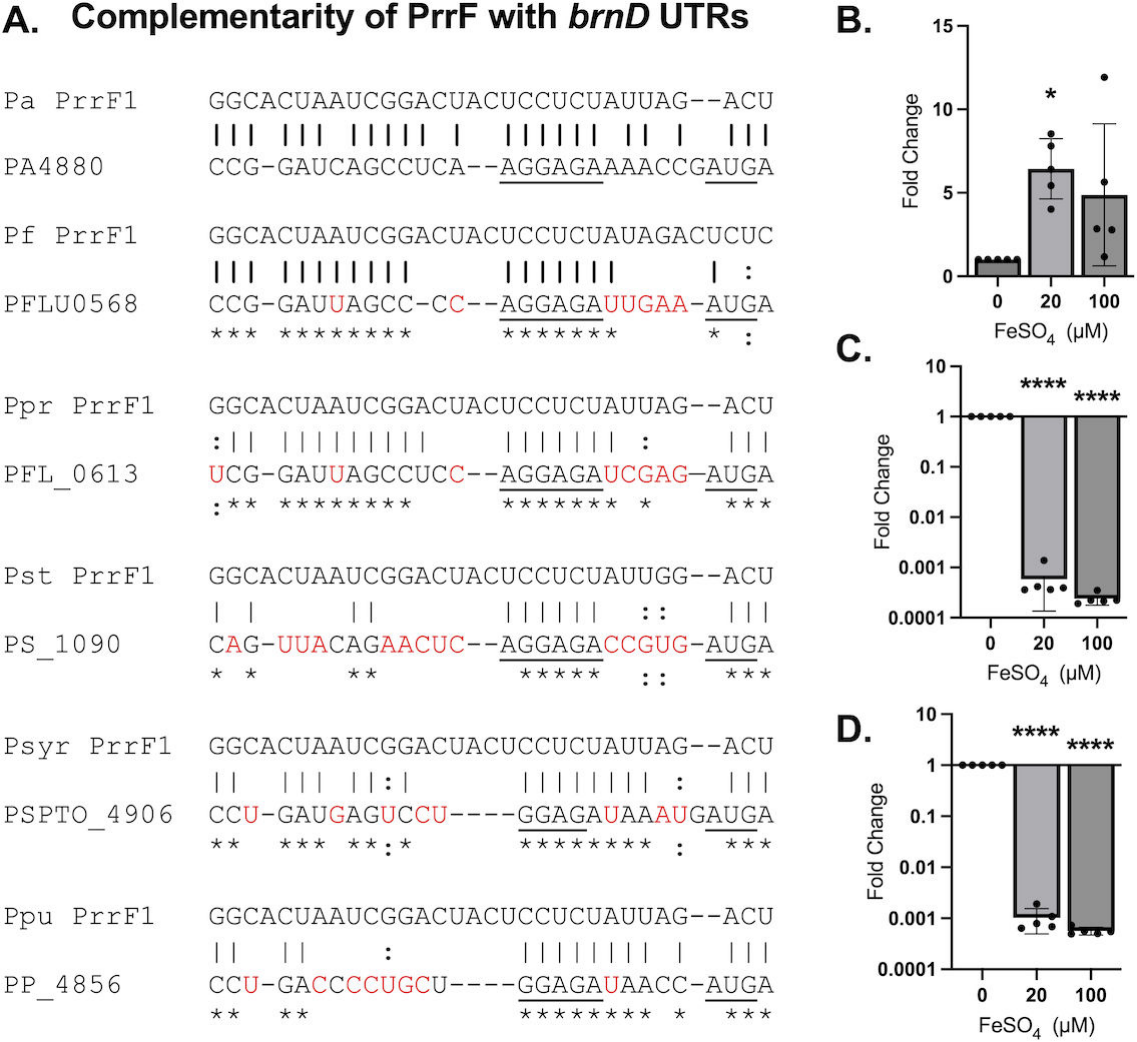
Iron regulation of *brnD* is conserved in Pf0-1. (**A**) Complementarity of PrrF homologs in the indicated *Pseudomonas* species (*Pa*, *P. aeruginosa*; *Pf*, *P. fluorescens*; *Ppr*, *P. protegens*; *Pst*, *P. stutzeri*; *Psyr*, *P. syringae*; *Ppu*, *P. putida*) with the cognate *brnD* 5′ UTR sequence. (B–D) Pf0-1 was grown shaking for 18 h in CDM supplemented with or without 20–100 µM FeSO_4_. Cultures were harvested for RNA and qPCR analysis of the native *brnD* mRNA (**B**), PrrF1 (**C**), or PrrF2 (**D**) sRNAs. Statistics were performed using one-way analysis of variance (ANOVA) with Dunnett’s multiple comparisons test for significance: ****P < 0.0001 and *P < 0.05.

The *brnD* gene is highly conserved in the pseudomonads (Fig. S1), and sequence analysis of the *brnD* 5′ UTR and PrrF sRNAs from multiple *Pseudomonas* species shows that complementarity of each PrrF1 sRNA to its cognate *brnD* mRNA is also broadly conserved (Fig. 7A). We therefore investigated if iron regulation of *brnD* occurs in other pseudomonads. For this, we grew *P. fluorescens* strain Pf0-1 with shaking for 18 h in CDM, and we observed that supplementation of the medium with either 20 or 100 µM FeSO_4_ resulted in robust induction of *brnD* by qPCR (Fig. 7B). To determine if the increase in *brnD* expression upon iron supplementation correlated with repression of the Pf0-1 PrrF sRNAs, we next examined Pf0-1 PrrF expression by qPCR. Unlike in *P. aeruginosa*, where the PrrF sRNAs share 96% identity and cannot be differentiated by qPCR, the PrrF1 and PrrF2 sRNAs in *P. fluorescens* share only 80% homology (Fig. S6A). This allowed us to generate primers specific to each *P. fluorescens* PrrF sRNA (Fig. S6C; Table S2). Consistent with what we observed in *P. aeruginosa*, iron supplementation significantly reduced the levels of both PrrF sRNAs in *P. fluorescens* (Fig. 7C and D). Although iron regulation of *brnD* has previously been observed in this environmental species (45–47), to our knowledge, this is the first report showing iron regulation of the PrrF sRNAs in *P. fluorescens*.

### Peroxide stress increases levels of the PrrF sRNAs in *P. fluorescens*

**Fig 8:**
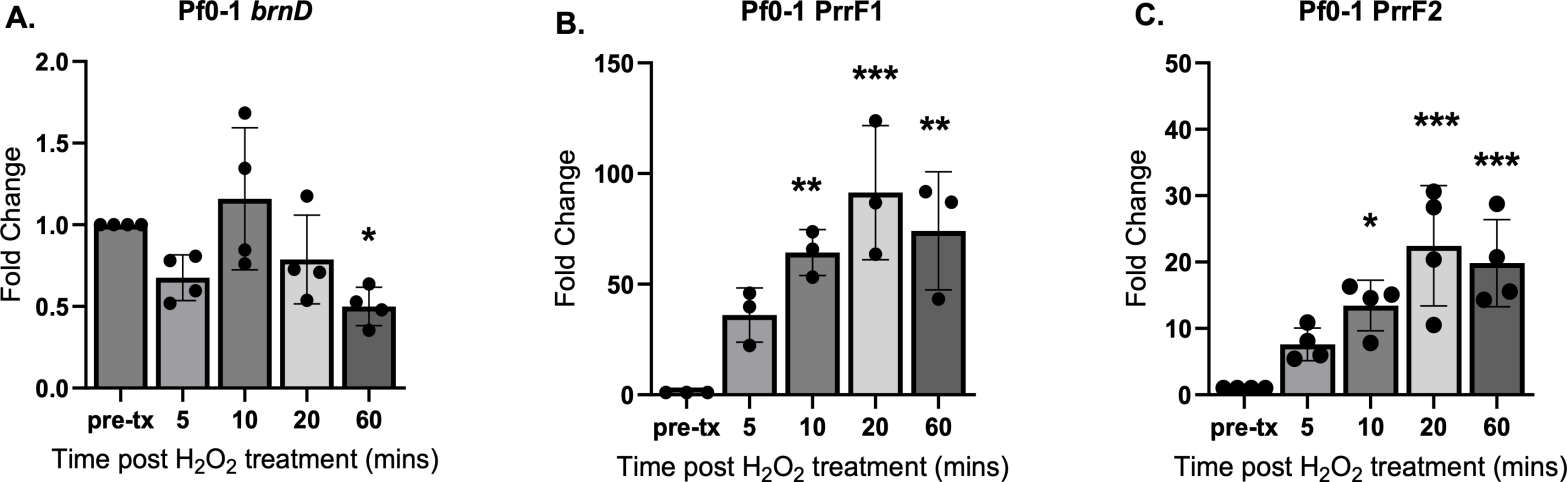
PrrF sRNAs are induced by peroxide treatment in Pf0-1. (**A–C**) Cultures of the Pf0-1 strain grown statically and harvested for qPCR analysis of the *brnD* mRNA (**A**), PrrF1 (**B**), or PrrF2 (**C**) sRNAs. Statistics were performed using one-way ANOVA with Dunnett’s multiple comparisons test for significance ***P < 0.0005, **P < 0.005, and *P < 0.05.

We next determined whether H_2_O_2_ treatment exerts a similar effect on PrrF and *brnD* expression in Pf0-1 as was observed in *P. aeruginosa*. For this, we grew Pf0-1 for 10 h in CDM with 20 µM iron supplementation, treated the cultures with 5 mM H_2_O_2_, and isolated RNA for qPCR analysis. Unlike what we observed in *P. aeruginosa*, *brnD* mRNA levels remained unchanged after the addition of peroxide to *P. fluorescens* cultures (Fig. 8A). In contrast, PrrF1 and PrrF2 transcript levels were both significantly induced for up to 60 min post-peroxide treatment (Fig. 8B and C). These data suggest that the increase in PrrF expression upon peroxide treatment is conserved among the pseudomonads.

## DISCUSSION

In this study, we investigated the role of the PrrF sRNAs in the regulation of genes involved in oxidative stress protection, with a focus on the recently crystallized protein BrnD (17). We showed that the PrrF sRNAs negatively affect *brnD* expression in *P. aeruginosa*, and this regulation is dependent upon the 5′ UTR of the *brnD* mRNA (Fig. 1A). The expression of *brnD* was also induced by peroxide treatment, despite increased expression of the PrrF sRNA levels (Fig. 1B). Moreover, we observed iron regulation of *brnD* and the PrrF sRNAs in *P. fluorescens*, and peroxide treatment similarly resulted in increased levels of the PrrF sRNAs. Notably, *brnD* expression was unaffected by peroxide treatment in *P. fluorescens* (Fig. 8A). Taken together, these data suggest that BrnD plays a role in the oxidative stress response of *P. aeruginosa*, and PrrF sRNAs present an intriguing role in the oxidative stress responses of both *P. aeruginosa* and *P. fluorescens*.

Our studies with oxidative stress treatment suggest that PrrF regulatory activity is hindered as cells manage their response to oxidative stress. This hindrance could be due to another regulatory factor sequestering either PrrF or the target mRNAs, or alternatively, due to inactivity of accessory factors that are required for PrrF-mediated gene regulation. For example, host-factor Q (Hfq) is a chaperone protein that is important for the recruitment of the PrrF sRNAs and has at least one mRNA target, *antR* (48). It would not be surprising if oxidative stress affected Hfq’s interaction with other macromolecules, resulting in the prolonged increased mRNA levels of *sodB* and *brnD*, even in the presence of PrrF. Alternatively, increased transcription of *sodB* and *brnD* upon peroxide treatment may overcome the impacts PrrF-mediated regulation of these mRNAs. Importantly, the mechanism by which *sodB* expression is induced upon peroxide treatment remains unclear, as the SoxRS regulatory system that activates *E. coli sodB* in response to oxidative stress is not involved in the *P. aeruginosa* oxidative stress response (49–52). Understanding how cells mitigate the impact of increased PrrF sRNA levels as cells manage their response to oxidative stress is a needed direction for future studies.

It also remains unclear whether *prrF1* promoter activity is indeed increased upon peroxide treatment, or if our data are instead due to differential impacts of oxidative stress on expression of PrrF2 versus PrrF1. If there are indeed differential effects of oxidative stress on the expression of each sRNA, it would be intriguing to determine whether this is due to PrrF2-specific regulatory proteins, such as AlgR (53, 54). AlgR is a regulatory protein that binds to the *algD* promoter to activate expression of genes for alginate production, leading to the mucoid phenotype characteristic of chronic *Pseudomonas* infections in the CF lung (55). AlgR also induces several genes involved in oxidative stress protection (56), and the production of alginate protects *P. aeruginosa* biofilms against oxidizing agents (57). AlgR additionally binds to the *pvdS* promoter (53), which is also repressed by Fur, and we observed a similar significant induction of *pvdS* upon H_2_O_2_ treatment in the *prrF2* transcriptional reporter strain (Fig. S5). One intriguing model is that AlgR binding to the *prrF2* promoter upon oxidative stress competes with the more limited pool of active Fur protein, resulting in enhanced *prrF2* promoter activity.

Recent structural and functional characterization of BrnD revealed that this protein has the capacity to store iron in the cavity of the 12-mer Dps fold (17). This work further showed that BrnD, like Dps (PA0962), binds to plasmid DNA *in vitro* (17, 27), suggestive of BrnD playing a role in oxidative stress protection of DNA. Given the potential links between BrnD and alginate, we predict that this protein plays a prominent role in biofilm physiology. BrnD also exhibits robust endonuclease activity (17), which the authors of this study posit plays a role in restriction protection against foreign DNA and/or phage—an activity that would be highly relevant to biofilm communities (58, 59). We are currently pursuing biofilm studies with a PAO1 ∆*brnD* mutant to understand its role in this important growth condition.

The extension of our studies into a non-pathogenic pseudomonad revealed conservation of iron regulation of PrrF and *brnD*, and they further showed a similar induction of PrrF upon oxidative stress. Although *P. fluorescens* is non-pathogenic and does not encounter host-derived oxidative stress, this organism is still susceptible to the deleterious effects of oxidizing agents. Aerobic respiration is one of the main causes of ROS production (43), and auto-oxidation of cellular enzymes can lead to elevated levels of intracellular peroxide (60, 61). *P. fluorescens* possesses most of the compendium of the alginate regulatory and biosynthetic genes, and alginate aids in *P. fluorescens* adaptation to osmotic stress from drier soil environments (62). Alginate also protects *Pseudomonas putida* biofilms against ROS and osmotic stress (63, 64). We have identified a conserved region of the AlgR-binding site upstream of the *P. fluorescens prrF1* and *prrF2* sequences (Fig. S7C and D), extending the potential for testing the AlgR-Fur competition model to this environmental pseudomonad. Future studies into how AlgR affects peroxide-induced expression of the PrrF sRNAs may therefore reveal novel paradigms for oxidative stress responses across the pseudomonads.

## MATERIALS AND METHODS

### Bacterial strains, media, and conditions

Strains used in this study are listed in Table S1. *P. aeruginosa* was grown in a previously described CDM (65–67). CDM was prepared as previously described (66) and supplemented with 1 mM CaCl_2_, 0.1 µM CuCl_2_, 0.1 µM NiCl_2_, 6 µM ZnCl_2_, and 0.3 µM MnCl_2_, and with or without 20 or 100 µM FeSO_4_, as specified, to afford metal-replete CDM. *P. aeruginosa* lab strains of PAO1 and their deletion mutants were routinely grown overnight by streaking from freezer stocks in tryptic soy agar or brain heart infusion agar (Sigma, St. Louis, MO) plates and incubated at 37°C for 12–16 h. Three to five colonies were taken from each streaked plate and inoculated in 1.5 mL of tryptic soy broth or LB Broth (Miller; Sigma, St. Louis, MO) and incubated for 12–16 h with shaking at 250 rpm at 37°C. Shaking cultures were grown aerobically in 5 mL of metal-replete CDM in 50 mL acid-washed glass flasks with foam stoppers. Cultures for growth assays were inoculated to an OD_600_ of 0.05 and grown for labeled timepoints, with shaking at 250 rpm at 37°C. Static cultures were grown micro-aerobically in 2 mL of high-iron, metal-replete CDM in 15 mL plastic culture tubes. Cultures for growth assays were inoculated to an OD_600_ of 0.05 and grown for 10 h, growing unperturbed in a standing incubator at 37°C. All experiments were performed with at least three biological replicates to ensure reproducibility.

### Generation of PAO1 *brnD* translational reporter strain

The UTR of *brnD* was amplified by PCR using primers mentioned in Table S2 (*brnD* UTR For and Rev) and the PAO1 genomic DNA as a template. The PCR product was digested by restriction enzymes BamHI and HindIII and directly cloned into the multiple cloning site of the P*_lac_*-mini-CTX-GFP^-SD^ vector (Table S1). This reporter plasmid lacks the *lacZ* Shine-Dalgarno site and was digested by the same restriction enzymes used for the UTR fragment to generate *P_lac_-brnD* UTR*::gfp^-SD^* plasmid for translational reporter assays. The reporter construct was then introduced into PAO1 and Δ*prrF* chromosomes by integrating them at the CTX *attB* site as previously described (68).

### Generation of PAO1 P*_prrF2_*::*gfp* reporter strain

The promoter region of *prrF2* gene (P*_prrF2_*) was amplified by PCR and the PAO1 genomic DNA as a template. The vector pMQ37 containing the coding region for green fluorescent protein was linearized by digestion with restriction enzyme HindIII. The linearized vector and P*_prrF2_* PCR product were transformed into yeast (*Saccharomyces cerevisiae* INVSc1), where the P*_prrF2_* PCR product was incorporated into the pMQ37 vector via homologous recombination. The pMQ37-P*_prrF2_* plasmid was isolated from yeast culture and transformed into *E. coli* strain SM10λ. The pMQ37-P*_prrF2_* plasmid was isolated and digested by restriction enzymes BamHI and EcoRI to clone them into the reporter plasmid mini-CTX-1 to generate mini-CTX-P*_prrF2_*-GFP plasmid. The vector was digested by the same restriction enzymes used for the promoter fusion fragment. The mini-CTX-P*_prrF2_*-GFP plasmid was transformed in *E. coli* strain SM10λ. The mini-CTX-P*_prrF2_*-GFP plasmid was isolated and transformed into *P. aeruginosa* PAO1 via electroporation. pFLP plasmid was isolated and transformed into PAO1 mini-CTX-P*_prrF2_*-GFP via electroporation to excise the integrated mini-CTX after the P*_prrF2_*-GFP reporter construct was introduced into PAO1 at the chromosomal *att* site as previously described (68).

### Beta-galactosidase reporter assays

The *brnD* reporter construct in PAO1 WT and *∆prrF* backgrounds was assayed for beta-galactosidase activity. Strains were inoculated to an OD_600_ of 0.05 into CDM supplemented with 1 mM CaCl_2_, 0.1 µM CuCl_2_, 0.1 µM NiCl_2_, 6 µM ZnCl_2_, and 0.3 µM MnCl_2_. In addition, 20 or 100 µM FeSO_4_ was supplemented for high iron cultures and grown shaking or static as described above at 37°C. Cells were then aliquoted at the listed timepoints and harvested by centrifugation, resuspended in potassium phosphate buffer, then diluted 1:10 in Z buffer (a buffer comprised of mono- and di-basic sodium phosphate, potassium chloride, magnesium sulfate, and fresh *β*-mercaptoethanol added to each aliquot); chloroform and 1% SDS were also added into the reaction mixture. o-Nitrophenyl-β-D-galactopyranoside was then used as a substrate to begin the reaction, which was stopped by the addition of 1 M Na_2_CO_3_, once sufficient yellow color was observed. The OD_420_ was measured for each sample, and the beta-galactosidase activity was calculated using the miller units formula = (1,000 × OD_420_)/(time × volume × OD_600_).

### GFP reporter assays

The *brnD* translational reporters and the PrrF promoter reporter strains were assayed for GFP fluorescence. Three to five replicates of the PAO1 and Δ*prrF* reporter strains were grown statically for 10 h as described above in either 0, 20, or 100 μM FeSO_4_ supplemented MR CDM. After 10 h, samples were either treated with 5 mM H_2_O_2_ or not, and 200 μL of each sample was aliquoted into black-bottom 96-well plates (Thermo-Fisher Scientific). GFP fluorescence was obtained by exciting the samples at 485 nm and emitting at 528 nm, with the laser set to a gain of 100. The OD_600_ readings of the samples were also obtained, to which the fluorescence readings were normalized.

### Real-time PCR

Five cultures of Pf0-1 or PAO1 and the isogenic *∆prrF* mutant were grown as previously described in MR CDM, either in shaking or static conditions, as indicated. RT-PCR was performed as previously described (13). Briefly, harvested cultures were stored in RNA later at −80°C. RNA was extracted using the Qiagen RNeasy kit, cDNA was synthesized, and RT-PCR was performed using TaqMan reagents (Roche) and a StepOnePlus system (Thermo-Fisher) for PAO1 strains and SYBR Green reagents and a QuantStudio system (Thermo-Fisher). Standard curves were produced for each primer/primer-probe set listed in Table S1 in the supplemental material by analyzing cDNA generated from serial dilutions of RNA and used to determine relative amounts of RNAs as described previously (13). Relative RNA levels were then normalized to the levels of 16S ribosomal RNA in respective strains.

### Statistical analysis

Statistical testing is done using Prism 9. Two-way analysis of variance with Tukey’s test for multiple comparisons (unless otherwise stated) was used to analyze the statistically significant changes in reporter assays and real-time PCR experiments using at least three biological replicates with a significance threshold of *P* value < 0.05.

## SUPPLEMENTAL MATERIAL

Supplemental materials jb.00534-25-s0001.pdf Tables S1 and S2, Figures S1 to S7, and Supplemental references.

ASM does not own the copyrights to Supplemental Material that may be linked to, or accessed through, an article. The authors have granted ASM a non-exclusive, world-wide license to publish the Supplemental Material files. Please contact the corresponding author directly for reuse.
