# Supplementary material for: Iron and peroxide regulation of the PrrF sRNAs and a conserved ferritin family protein in Pseudomonas aeruginosa and Pseudomonas fluorescens
Source: J Bacteriol. 2026 Mar 30;208(4):e00534-25. doi: 10.1128/jb.00534-25 (PMC13104616; doi:10.1128/jb.00534-25)
Supplement: Supplemental materials — Tables S1 and S2, Figures S1 to S7, and Supplemental references. [file jb.00534-25-s0001.pdf]

Supplementary materials for:

Iron and peroxide regulation of the PrrF sRNAs and a conserved ferritin family protein in *Pseudomonas aeruginosa* and *Pseudomonas fluorescens*

Khady O. Ouattara[1], Rhishita Chourashi[1], and Amanda G. Oglesby[1,2]

University of Maryland, Baltimore, [1]School of Pharmacy, [1]Department of Pharmaceutical Sciences and [2]School of Medicine, Department of Microbiology and Immunology, Baltimore, Maryland, 21201

Running Title: Oxidative stress alters PrrF regulation

Keywords: PrrF, BrnD, *Pseudomonas aeruginosa*, *Pseudomonas fluorescens*, iron, oxidative stress

*To whom correspondence should be addressed: aoglesby@rx.umaryland.edu

Table of Contents

Table S1. Strains and plasmids used in this study 3

Table S2. Primers and probes used in this study 4

Figure S1. BrnD ortholog alignment shows conservation of residues 5

Figure S2. Iron titration of *brnD* reporter shows minimum high iron concentration 6

Figure S3. PrrF regulation at *brnD*'s UTR is masked in shaking conditions 7

Figure S4. RT-PCR show PrrF regulation of *sodB* and not *katA* in static conditions 7

Figure S5. AlgR target pyoverdine sigma factor is upregulated in *prrF2* promoter reporter strain 8

Figure S6. Sequence alignments show *prrF1,2* sequence conservation 8

Figure S7. Sequence analysis reveals possible AlgR binding site upstream of Pf0-1 PrrF sRNAs 9

Supplemental References. 10

Table S1. Strains and plasmids used in this study

Strain/Plasmid	Description	Reference
Strains		
PAO1	Human wound isolate originally isolated in Australia	(1)
PAO1 Δ*prrF*	*prrF1,2* deletion in PAO1	(2)
Pf0-1	Free-living environmental strain isolated from agricultural loam (sand, clay, and organic matter) soil in 1988 by Compeau *et al*. and is well adapted to soil environments.	(3)
Plasmids		
pMQ37 (pLD2477)	MCS-gfp flanked by upstream and downstream 1 kb genomic sequences at *attB* site was inserted in pMQ30 through gap repair cloning	(4)
SM10 / pFLP	SM10 carrying the pFLP recombinase	(5)
P_{brnD}:brnD5'UTR:*lacZ*	PAO1 with the full brnD reporter fusion containing P_{brnD} and *brnD*'s 5'UTR fused to the lacZY gene and integrated at the chromosal att site	This study
P_{prrF1}:*gfp*	Mini-CTX with the P_{prrF1}::*gfp* reporter fusion containing the *prrF1* promoter fused to the *gfp* Shine-Delgarno sequence and coding region	(6)
P_{prrF2}:*gfp*	Mini-CTX with the P_{prrF2}::*gfp* reporter fusion containing the *prrF1* promoter fused to the *gfp* Shine-Delgarno sequence and coding region	This study
P_{lac}:*gfp*$^{-SD}$	Mini-CTX containing the P_{lac} constituitive promoter and the Shine-Delgarno site deleted fused to the *gfp* gene; adapted from the mini-CTX1:P_{lac}:'lacZ^{-SD} plasmid	(7)
P_{lac}:*brnD*5'UTR:*gfp*$^{-SD}$	PAO1 containing the P_{lac} constituitive promoter and *brnD*'s 5'UTR region fused to the *gfp* gene integrated at the chromosomal att site	This study

Table S2. Primers and probes used in this study

**Name**	**Sequence (5' to 3')**	**Reference**
16S Forward	GCGTAGGTGGTTCAGCAAGT	(8)
16S Reverse	CATTTCACCGCTACACAGGA	(8)
16S Probe	ACTGAGCTAGAGTACGGTAGAGGGTGGTGG	(8)
prrF Forward	AACTGGTCGCGAGATCAGC	(2)
prrF Reverse	CCGTGATTAGCCTGATGAGGAG	(2)
prrF Probe	CCCACGCAGTCGGACTCTTCAGATT	(2)
brnD Forward	CTCGCCACCGAGTTG	This study
brnD Reverse	ACGTATTCGGCATGGG	This study
brnD Probe	TCGAGCACGCCAACCAGGAAATG	This study
gfp Forward	CACTACTTTGACTTATGGTGTTC	This study
gfp Reverse	GTCTTATAGTTCCCGTCATCTT	This study
gfp Probe	TTTCAAGAGTGCCATGCCCGAAGG	This study
katA Forward	TGTTCTATCTGCGCGACCC	This study
katA Reverse	TCCCACTTGAAGGTCGCATT	This study
katA Probe	ATCTCAACCACGTGGTGAAGC	This study
sodB Forward	AACACCTACGTGGTGAACCTGA	(9)
sodB Reverse	TGACGATCTCTTCGAGGCTCTT	(9)
sodB Probe	CCTGATCCCGGGCACCGAGTT	(9)
Pf0-1 16S Forward	AGCTACTTGGTGAGGTAATG	This study
Pf0-1 16S Reverse	CAGTTCCAGTGTGACTGAT	This study
Pf0-1 *prrF1* Forward	GATCGCGAGATCAGTCGATATT	This study
Pf0-1 *prrF1* Reverse	GTGATTAGCCTGATGAGGAGATAA	This study
Pf0-1 *prrF2* Forward	CTGGTCGCGAGATCAGTC	This study
Pf0-1 *prrF2* Reverse	GTGATTAGCCTGATGAGGAGATAG	This study
Pf0-1 *brnD* Forward	GCGGTGACCGAAAGC	This study
Pf0-1 *brnD* Reverse	GTAGTGGCGCTTGTAGC	This study
brnD UTR For	GCGCGGATCCGGTAAGCAGAAAAAGCCAGCCGCAGA	This study
brnD UTR Rev	GCGCAAGCTTCAGTTGAACGGTGGTCATCGGTTTTCTC	This study
PrrF2 promoter For	ATGGAATGAATGAGAACCGGCT	This study
PrrF2 promoter Rev	TGAGAATAGTTATTATTACAC	This study

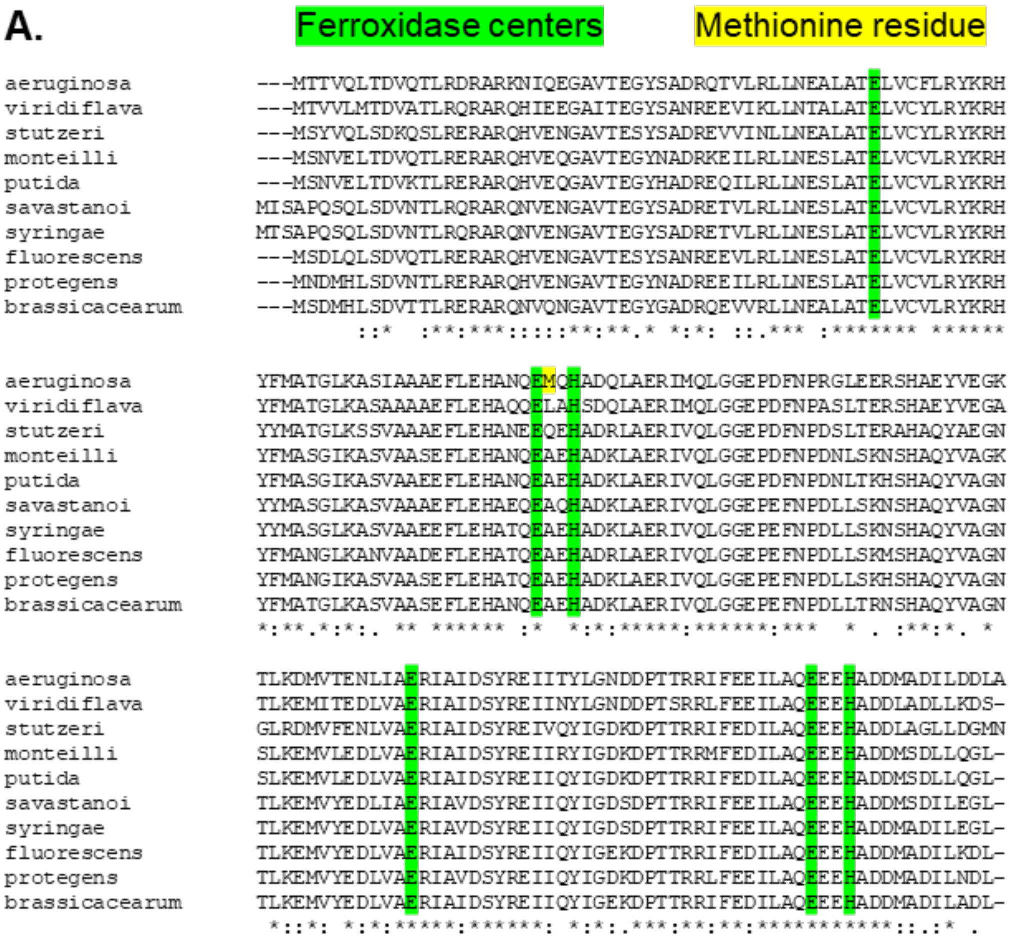

Figure S1. **BrnD ortholog alignment shows conservation of residues.** Sequence alignment of BrnD's protein sequence in ten *Pseudomonas* species, ranging from pathogenic to environmental. Highlighted in green are the catalytic ferroxidase center residues, and the heme-coordinating methionine only present in *P. aeruginosa* is shown in yellow.

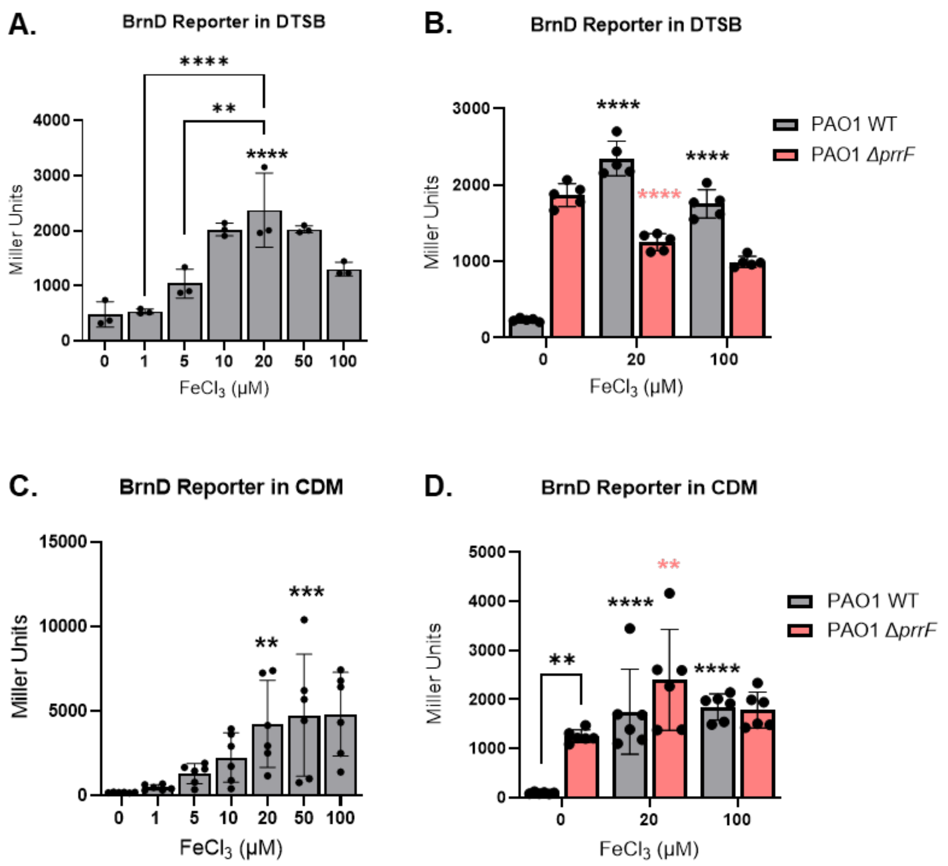

Figure S2. Iron titration of *brnD* reporter shows minimum high iron concentration. PAO1 (grey bars) carrying the *brnD* reporter construct (**Fig. 1A**) was grown with shaking (250 rpm) in DTSB (**A**) or CDM (**C**) with supplementation of the indicated concentrations of $FeCl_3$ for 18 h and assayed for β-galactosidase activity**.** PAO1 and the isogenic Δ*prrF* mutant (pink bars) strains carrying the *brnD* reporter were grown with shaking (250 rpm) in DTSB (**B**) or CDM (**D**) with supplementation of the indicated concentrations of $FeCl_3$ for 18 h and assayed for β-galactosidase activity. Statistics were performed using two-way ANOVA, with Tukey's multiple comparisons test for significance. Floating asterisks represent strain-specific (black for wild-type and pink for Δ*prrF*) significance in reference to 0 μM $FeSO_4$, to which all conditions were normalized: ****$P<0.0001$, ***$P<0.0005$, **$P<0.005$, and *$P<0.05$.

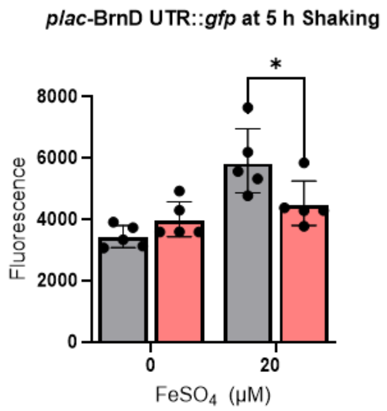

Figure S3. **PrrF regulation at *brnD*'s UTR is masked in shaking conditions.** Wild-type (gray bars) and Δ*prrF* (pink) strains carrying the *brnD* translational reporter (**Fig. 3A**) were grown statically for 5 h in CDM with or without supplementation of 20 μM $FeSO_4$. GFP was excited at 485nm and fluorescence emitted at 528nm, with a gain of 100. Statistics were done using two-way ANOVA, with uncorrected Fisher's LSD for significance: *P<0.05.

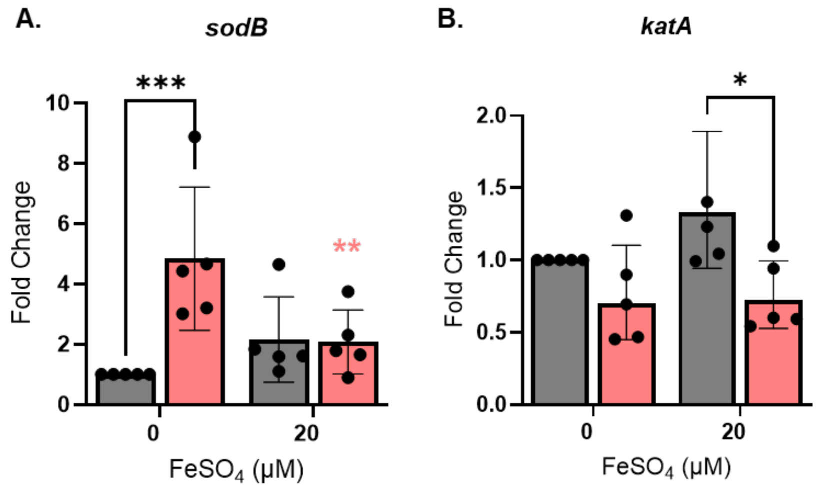

Figure S4. **RT-PCR show PrrF regulation of *sodB* and not *katA* in static conditions.** PAO1 and the isogenic Δ*prrF* mutant (pink bars) were grown statically in CDM with or without supplementation of 20 μM $FeSO_4$ were grown statically for 10 h and RNA was isolated for qPCR analysis of the *sodB* mRNA (**A**) and the *katA* mRNA (**B**). Statistics were performed using two-way ANOVA, with Tukey's multiple comparisons test for significance. Floating asterisks represent strain-specific significance (black asterisks for wild-type and pink asterisks for Δ*prrF*) in reference 0 μM $FeSO_4$ (**B-D**): ***P<0.0005, **P<0.005, and *P<0.05.

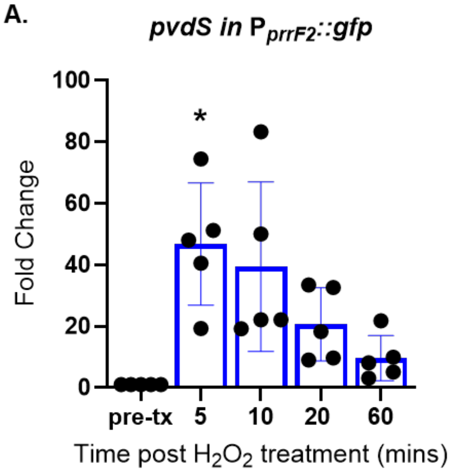

Figure S5. AlgR target *pvdS* is upregulated in *prrF2* promoter reporter strain. PAO1 cultures carrying the P_{prrF2}::*gfp* promoter reporter were grown statically for 10 h in CDM supplemented with 20μM $FeSO_4$ then treated with 5 mM H_2O_2. Aliquots were collected and harvested for RNA isolation and qPCR analysis of the *pvdS* mRNA at the indicated timepoints post-treatment. Statistics were performed using One-way ANOVA, with Dunnett's multiple comparisons test for significance: *P<0.05.

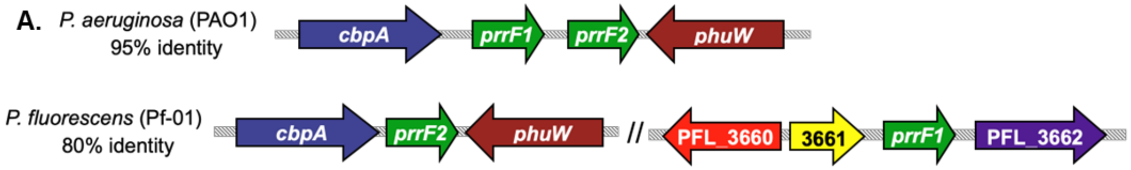

B. *P. aeruginosa* PrrF1 and PrrF2

```
PAO1_PrrF1      AACTGGTCGCGAGATCAGCCGGTAAGCTGAGAGACCCACGCAGTCGGACTCTTCAGATTA     60
PAO1_PrrF2      AACTGGTCGCGAGGCCAGCAGGTAAGCTGAGAG-ACCAAGCAGTCGGACTCTTCAGATTA     59
                *************  **** *************  *** *********************

PAO1_PrrF1      TCTCCTCATCAGGCTAATCACGGTTTTTGACCCGGCACTTTGCCGGGTCTTTTTTT     116
PAO1_PrrF2      TCTCCTCATCAGGCTAATCACGGTTTCGACCCG-GCACTTTGCCGGGTCTTTTTTT     114
                **************************    **  **********************
```

C. *P. fluorescens* PrrF1 and PrrF2

```
Pf0-1_PrrF1     AACTGATCGCGAGATCAGTCGATATTCTGAAAAGCCCTTGGTTCGGACTCTCAGATTATC     60
Pf0-1_PrrF2     AACTGGTCGCGAGATCAGTCGATG--GACCAGAGACCTTAGGTCGGTCTTCTGGACTATC     58
                ***** *****************       * ** **** * **** **    ** ****

Pf0-1_PrrF1     TCCTCATCAGGCTAATCACGGTTATTTGACCCGGTTTTTACCGGGTCTTTTTTT     114
Pf0-1_PrrF2     TCCTCATCAGGCTAATCACGGTTTTTGACCCGGCTTTTTGCCGGGTCTTTTTTT     112
                *********************** **   ** * ***** **************
```

Figure S6. Sequence alignments show *prrF1,2* sequence conservation. A. Genomic context of the arrangement of the *prrF1* and *prrF2* loci in *P. aeruginosa* (top) and in *P. fluorescens* (bottom) and their sequence identity percentage. **B-C.** DNA sequence alignment of *prrF1* and *prrF2* in *P. aeruginosa* (**B**) and in *P. fluorescens* (**C**), showing the conservation of nucleotides (asterisks) and the primers used to amplify the sequences for qPCR. PrrF1 primers are shown in bold red and PrrF2 primers are shown in bold red in 5' to 3' order.

A. AlgR binding consensus sequence

A**CCGTT**CGTC

B. *P. aeruginosa* PrrF2

GCCGTGTTCACTCGCGAGAAACGCAGCGCCACGGTGGTCGCCAGCGAACCCTGCACGGTGATGGTGATTCCCAAGGA
GCAGTTCCTCGGCCTTATGCAGAACAACCCGCGAATCGCCCATAGCCTGATCGAAGGCATGGCCCGGCGGATAGACC
TGCTTAACCGGGAAGTGACCCAGCTGCGCCTGCGCCGCAGCGCCTGAGGCCCATTCCAGAGGGCTCGCGACTAGCTA
GCAGAAAAGTTTGGCGAAAGCGTTTGACATGGAAATGAGAATCATTATTATGTCACTCAACTGGTCGCGAGATCAGC
CGGTAAGCTGAGAGACCCACGCAGTCGGACTCTTCAGATTATCTCCTCATCAGGCTAATCACGGTTTTTGACCCGGC
ACTTTGCCGGGTCTTTTTTTGCCTGCGATTCGGCCGGAGACG[**ACCGTTC**ATCG]GCTGGCGATGGAATGAATGAGA
ACCGGCTTGACCTGATAATGAGAATAGTTATTATTACACCAACTGGTCGCGAGGCCAGCAGGTAAGCTGAGAGACCA
AGCAGTCGGACTCTTCAGATTATCTCCTCATCAGGCTAATCACGGTTTCGACCCGGCACTTTGCCGGGTCTTTTTTT

C. *P. fluorescens* PrrF1

CACTACTGGCGCACGCGGTAGCCGAGTTCAAGCCGCGTGAGTTTCGCAGCACCAATGGTTTCAAGCGGGTCGACGCC
GGAGACATTCTGATCCGCGAGGGCGATGACGCCGACCATGTGTTCGTGATTATCGAAGGACACGCCGAAGCGTTTGT
TAACGGGCACAAGGTAGGCGATGTGCCCAAGGATGAGATATTCGGCGCAATG[G**CCGT**GTTCAC]TGGCGAGCCGCG
CAACGCAACGGTCATTGCCCGGGAAGCGAGCACGGTGATGCTGATTCCCGGCGATCAGTTTCTGAGCATGACCCGCA
CCAATCCGAAAATCGCCCACAGCCTGATCGAAAGCATGGCGCTGCGTATCGACCAGCTCAACAAGCAGCTCACGGGA
TTTAACAGCCAGACCTGACGCCGGTATTCACGGGGCGTTTACGCCATTGCCGGGACAAAACGACAGAAATGAAAAAA
CAGCAGTTGACTTGGTAATGAGAATCGCTATGATTATCACAACTGATCGCGAGATCAGTCGATATTCTGAAAAGCCC
TTGGTTCGGACTCTCAGATTATCTCCTCATCAGGCTAATCACGGTTATTTGACCCGGTTTTTACCGGGTCTTTTTTT

D. *P. fluorescens* PrrF2

TCTGGTCACAAATACTATTGGTACTATTCTTGGGTCACGGATTTGAATCGACCAAGGAGTGACCTTTCACCATGAAT
ATCCCCAGCAAAGACTTGCCCGACCTGCAAATGGACACCACCTTTACGTCTCCGCAAGGGTGCGCCGCTGCGCAACG
GGCGTTGGACTATTACTTGAAACCAGCTGTGTCAGAACCGGAAGTGGATGAGCGCTTTTTCGGCGTGAACAGCAACC
TGAGTGGCGAAGAATCCCTGGTCCACGCCTCCGACCTGC[T**CCGTT**GTGCA]GCAGCGACGGCGTTCAAGGCTGCGG
ACAGCTTGCAGGGCGTCAATCGGGATCTGGCGTTTTCGGTCGTGCACATGGTGGACATGGCGCGGGCGATGGTTGAT
CACTCGCTTGATGGCGCAGTGCGCTGAAGACGGGAGTTCGGACGGACAGGAAAGAACTTTCCTATCGCGCAGATAAA
AACATTTGACTTGCAAATGATAATGATTATTATTGCAAGCAACTGGTCGCGAGATCAGTCGATGGACCAGAGACCTT
AGGTCGGTCTTCTGGACTATCTCCTCATCAGGCTAATCACGGTTTTTGACCCGGCTTTTTGCCGGGTCTTTTTTT

Figure S7. Sequence analysis reveals possible AlgR binding site upstream of Pf0-1 PrrF sRNAs. A. Shows the conserved core sequence of the AlgR binding site, found in the upstream region of *algD* (10–13). **B-D**. 11 base pair (bp) AlgR binding site annotated in between brackets and underlined upstream of *P. aeruginosa prrF2* (**B**; DNA sequence in cyan), of *P. fluorescens prrF1* (**C**; DNA sequence in magenta), and of *P. fluorescens prrF2* (**D**; DNA sequence in yellow).

Supplementary Materials References

1. Holloway BW. 1955. Genetic Recombination in Pseudomonas aeruginosa. Microbiology 13:572–581.
2. Wilderman PJ, Sowa NA, FitzGerald DJ, FitzGerald PC, Gottesman S, Ochsner UA, Vasil ML. 2004. Identification of tandem duplicate regulatory small RNAs in *Pseudomonas aeruginosa* involved in iron homeostasis. Proc Natl Acad Sci 101:9792–9797.
3. Silby MW, Cerdeño-Tárraga AM, Vernikos GS, Giddens SR, Jackson RW, Preston GM, Zhang X-X, Moon CD, Gehrig SM, Godfrey SAC, Knight CG, Malone JG, Robinson Z, Spiers AJ, Harris S, Challis GL, Yaxley AM, Harris D, Seeger K, Murphy L, Rutter S, Squares R, Quail MA, Saunders E, Mavromatis K, Brettin TS, Bentley SD, Hothersall J, Stephens E, Thomas CM, Parkhill J, Levy SB, Rainey PB, Thomson NR. 2009. Genomic and genetic analyses of diversity and plant interactions of Pseudomonas fluorescens. Genome Biol 10:R51.
4. Lin Y-C, Sekedat MD, Cornell WC, Silva GM, Okegbe C, Price-Whelan A, Vogel C, Dietrich LEP. 2018. Phenazines Regulate Nap-Dependent Denitrification in Pseudomonas aeruginosa Biofilms. J Bacteriol 200:10.1128/jb.00031-18.
5. Hoang TT, Karkhoff-Schweizer RR, Kutchma AJ, Schweizer HP. 1998. A broad-host-range Flp-*FRT* recombination system for site-specific excision of chromosomally-located DNA sequences: application for isolation of unmarked *Pseudomonas aeruginosa* mutants. Gene 212:77–86.
6. Chourashi R, Weiner J, Hoang T-M, Ouattara K, Oglesby AG. 2024. The Pseudomonas aeruginosa PrrF sRNAs promote biofilm formation at body temperature. bioRxiv https://doi.org/10.1101/2024.12.11.628005.
7. Djapgne L, Panja S, Brewer LK, Gans JH, Kane MA, Woodson SA, Oglesby-Sherrouse AG. 2018. The Pseudomonas aeruginosa PrrF1 and PrrF2 Small Regulatory RNAs Promote 2-Alkyl-4-Quinolone Production through Redundant Regulation of the *antR* mRNA. J Bacteriol 200.
8. Weiner JM, Lee WH, Nolan EM, Oglesby AG. 2025. Calprotectin elicits aberrant iron starvation responses in Pseudomonas aeruginosa under anaerobic conditions. J Bacteriol 207:e0002925.
9. Reinhart AA, Nguyen AT, Brewer LK, Bevere J, Jones JW, Kane MA, Damron FH, Barbier M, Oglesby-Sherrouse AG. 2017. The Pseudomonas aeruginosa PrrF Small RNAs Regulate Iron Homeostasis during Acute Murine Lung Infection. Infect Immun 85:e00764-16.
10. Mohr CD, Leveau JH, Krieg DP, Hibler NS, Deretic V. 1992. AlgR-binding sites within the algD promoter make up a set of inverted repeats separated by a large intervening segment of DNA. J Bacteriol 174:6624–6633.
11. Ma J-F, Phibbs PV, Hassett DJ. 2006. Glucose stimulates alginate production and algD transcription in Pseudomonas aeruginosa. FEMS Microbiol Lett 148:217–221.
12. Okkotsu Y, Little AS, Schurr MJ. 2014. The Pseudomonas aeruginosa AlgZR two-component system coordinates multiple phenotypes. Front Cell Infect Microbiol 4:82.
13. Kong W, Zhao J, Kang H, Zhu M, Zhou T, Deng X, Liang H. 2015. ChIP-seq reveals the global regulator AlgR mediating cyclic di-GMP synthesis in Pseudomonas aeruginosa. Nucleic Acids Res 43:8268–8282.
